# Impact of obesity and epicardial fat on early left atrial dysfunction assessed by cardiac MRI strain analysis

**DOI:** 10.1186/s12933-016-0481-7

**Published:** 2016-12-22

**Authors:** Morgane Evin, Kathryn M. Broadhouse, Fraser M. Callaghan, Rachel T. McGrath, Sarah Glastras, Rebecca Kozor, Samantha L. Hocking, Jérôme Lamy, Alban Redheuil, Nadjia Kachenoura, Greg R. Fulcher, Gemma A. Figtree, Stuart M. Grieve

**Affiliations:** 1 Sydney Translational Imaging Laboratory, Heart Research Institute, Charles Perkins Centre, University of Sydney, Camperdown, NSW 2006 Australia; 2 Sydney Medical School, University of Sydney, Camperdown, Australia; 3 Sorbonne Universités, UPMC Univ Paris 06, INSERM UMRS 1146, CNRSUMR 7371, Laboratoire d’Imagerie Biomédicale, ICAN Institute of Cardiometabolism and Nutrition, Paris, France; 4 Department of Endocrinology, Royal North Shore Hospital, St Leonards, Australia; 5 Kolling Institute, University of Sydney, Sydney, Australia; 6 Department of Cardiology, Royal North Shore Hospital, St Leonards, Australia; 7 Departments of Radiology, Royal North Shore Hospital and Royal Prince Alfred Hospital, Sydney, Australia

**Keywords:** Diastolic dysfunction, Cardiac dysfunction, Magnetic resonance studies, Fat distribution, Obesity and type 2

## Abstract

**Background:**

Diastolic dysfunction is a major cause of morbidity in obese individuals. We aimed to assess the ability of magnetic resonance imaging (MRI) derived left atrial (LA) strain to detect early diastolic dysfunction in individuals with obesity and type 2 diabetes, and to explore the association between cardiac adipose tissue and LA function.

**Methods:**

Twenty patients with obesity and T2D (55 ± 8 years) and nineteen healthy controls (48 ± 13 years) were imaged using cine steady state free precession and 2-point Dixon cardiovascular magnetic resonance. LA function was quantified using a feature tracking technique with definition of phasic longitudinal strain and strain rates, as well as radial motion fraction and radial velocities.

**Results:**

Systolic left ventricular size and function were similar between the obesity and type 2 diabetes and control groups by MRI. All patients except four had normal diastolic assessment by echocardiography. In contrast, measures of LA function using magnetic resonance feature tracking were uniformly altered in the obesity and type 2 diabetes group only. Although there was no significant difference in intra-myocardial fat fraction, Dixon 3D epicardial fat volume(EFV) was significantly elevated in the obesity and type 2 diabetes versus control group (135 ± 31 vs. 90 ± 30 mL/m^2^, p < 0.001). There were significant correlations between LA functional indices and both BMI and EFV (p ≤ 0.007).

**Conclusions:**

LA MRI-strain may be a sensitive tool for the detection of early diastolic dysfunction in individuals with obesity and type 2 diabetes and correlated with BMI and epicardial fat supporting a possible association between adiposity and LA strain.

*Trials Registration* Australian New Zealand Clinical Trials Registry No. ACTRN12613001069741

## Background

Obesity and diabetes are strong risk factors for heart failure with preserved ejection fraction (HFpEF), a clinical syndrome that affects nearly half of patients with symptoms of heart failure [1]. Diastolic dysfunction is an asymptomatic condition leading to heart failure and is traditionally assessed by echocardiography. However, a reliable and sensitive method for the early identification of diastolic dysfunction is currently lacking [2–4]. The earliest manifestations of diastolic dysfunction are typically evident in altered left atrial dynamics or pulmonary inflow patterns [5]. The features represent are good target for early diastolic dysfunction detection and improved methods for capturing these physiological signs would therefore be of great clinical and scientific interest.

Obesity and type 2 diabetes mellitus (ObT2D) commonly co-exist and together they contribute to a wide range of metabolic complications; importantly, they are increasingly recognised to be major contributors to the global burden of cardiac disease. The paucity of reliable early, non-invasive detection of diastolic changes on ObT2D population is therefore a key limitation of our current clinical and research capacity.

The changes associated with the mid and late stages of diastolic dysfunction in individuals with obesity and T2D include a complex spectrum of adipose infiltration (both intra-myocardial and epicardial), fibrosis and electrophysiological alterations. Of these factors, intra-myocardial and epicardial fatty changes have received considerable attention. However, the relationship between cardiac fat and early dysfunction remains poorly understood. This deficit is largely attributable to the difficulties in detecting very early alterations in cardiac function.

LA functional and volumetric changes are amongst the first imaging changes seen in ObT2D, and therefore may be a useful marker of early disease [6–8]. Recent data shows that diabetes is an independent risk factor predictive of LA enlargement and dysfunction. Data has also linked epicardial fat to LA and ventricular functional changes [9], and to increased LA volume [10, 11]. A highly powered analysis using a Framingham cohort demonstrated that this association is independent of other measures of adiposity in men [11].

Although echocardiography is the generally accepted gold standard for measurement of atrial function and other diastolic functional or structural changes, in the setting of obesity the practical difficulties of imaging obese patients make routine acquisitions problematic. Cardiovascular magnetic resonance (CMR) may present a good alternative for reproducible, accurate and sensitive quantification of LA function. The recent development of feature tracking algorithms that can be applied in a semi-automated manner to CMR data permits myocardial strain measurements to be obtained from cine functional images [12–14]. CMR simultaneously offers a convenient and accurate way to quantify pericardial fat using anatomical images, or with specific fat quantification techniques such as Dixon [15].

In this study, we applied a novel MRI method for measuring atrial strain to detect subclinical evidence for diastolic dysfunction in a cohort of obese individuals with type 2 diabetes and no clinical evidence of heart failure. As a secondary aim, we assessed the association between abnormal atrial function and both intra-myocardial and epicardial fat.

## Methods

### Study population

The first 20 consecutive patients from the study entitled “The combined Effect of Liraglutide and Sleeve Gastrectomy on Metabolic, Cardiac, Neurological and Sleep Function in Obese Diabetes” (LIRASLEEVE; mean age 55 ± 8 years; 40% female) with a diagnosis of obesity and type 2 diabetes mellitus were recruited via the Department of Endocrinology, Diabetes and Metabolism at Royal North Shore Hospital, Australia. Inclusion criteria included BMI >30 kg/m^2^ and known T2D (HbA1c ranged from 7 to 10% at study entry). Subjects were excluded if there was a contraindication to MRI or weight in excess of 150 kg (MRI table limit). Nineteen age and gender matched controls (48 ± 13 years; 47% female) with no history of cardiac disease were recruited. The local ethics committee approved the study and all patients provided written consent.

### Cardiovascular magnetic resonance imaging

CMR data were acquired using a 1.5T Siemens Avanto scanner at North Shore Private Radiology, St Leonards, Australia. Cardiac volumes and left ventricle (LV) mass were quantified using 2-chamber (LVLA), 4-chamber (4CH), 3-chamber (LVOT) views and a short axis stack of cine SSFP images (TE: 1.5 ms, TR: 3.4 ms, 20 phases; flip angle: 45°, acquisition; FOV: 35 cm, slice thickness: 8 mm). 2-point Dixon data was also acquired (TR 6.7 ms, resolution: 1.2 mm, slice thickness: 4 mm, TE_2_: 2.4, 4.8 ms). One patient and one healthy volunteer were excluded from the analysis as the Dixon images were not available.

### Ventricular functional analysis

Region of interest analysis was performed using the Segment software (Medviso, Lund, Sweden) [16]. LV end-diastolic (EDV) and end-systolic volumes (ESV), indexed LV mass, LV stroke volume (SV) and LV ejection fraction (LVEF) were obtained from the short axis stack by manually contouring end-diastolic and end-systolic endocardial borders and end-diastolic epicardial borders from the base to the apex.

### Left atrial strain and volume analysis

The analysis and feature tracking algorithm has previously been described in details [14]. It was quantified on LVLA, 4CH, and LVOT views and averaged for a global analysis. After endocardial contour tracking, longitudinal strain was defined as the temporal variation of the length of the contour, and was calculated as [Slt = (Lt − Lo)/Lo], where Lo is the initial length of the contour and Lt is the length at time t. Radial motion fraction corresponded to the radial relative displacement of the considered segment towards the LA centre of mass, [Mr = (Mt − Mo)/Mo], where Mo is the initial radius, and Mt, the radius at time *t*.

**Fig. 1:**
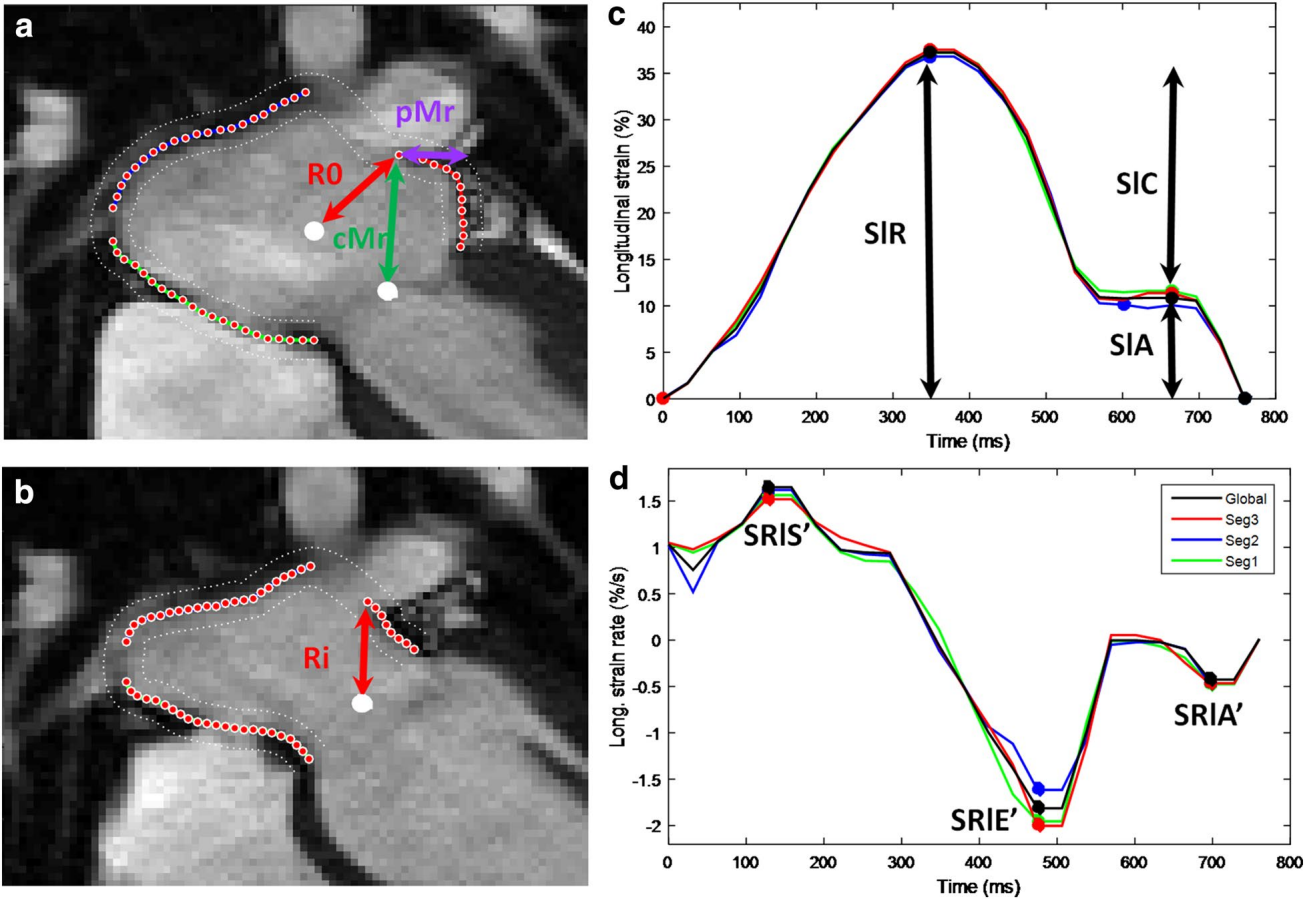
Illustration of the methodology for LA function analysis. **a**, **b** Left atrial contours from a 4-chamber view and longitudinal strain and strain rate related curves of a subject from the Obese-Type 2 diabetes (ObT2D) group. R_o_ example of a radius used for the computation of the radial motion fraction. Decomposition of the radial motion fraction into MV centric (cMr, towards the mitral valve center) and MV corrected perpendicular (pMr, MV corrected perpendicular radial motion fraction) components. **c**, **d** *Sl*_*R*_ reservoir longitudinal strain, *Sl*_*C*_ conduit longitudinal strain and *Sl*_*A*_ La contraction longitudinal strain, *SRL*_*S′*_ reservoir longitudinal strain rate, *SRl*_*E′*_ conduit longitudinal strain rate and *SRl*_*A′*_ LA contraction longitudinal strain rate

Figure 1 summarises the LA strain functional methodology and analysis. Briefly, parameters are classified according to reservoir (R), conduit (C) or atrial contraction (A) phases. LA phasic measurements were made for longitudinal strain (Sl) and radial motion fraction (Mr), where the phase is denoted as a suffix (Sl_R_, Sl_C_, Sl_A_), (Mr_R_, Mr_C_, Mr_A_) respectively. Longitudinal strain rates (SRl_S′_, SRl_E′_, SRl_A′_) and radial relative velocities (Vr_S′_, Vr_E′_, Vr_A′_) were computed, as the time derivatives of longitudinal and radial indices, for the three atrial functional phases: reservoir (S′), conduit (E′) and LA contraction (A′).

To further investigate the radial motion fraction modifications in term of LV related and LA standalone motions, this parameter was decomposed into two orthogonal components relative to the centre of the mitral valve (MV). The first, in the direction of the MV, was termed centric (cMr) and the other, normal to this direction (termed MV perpendicular: pMr, see Fig. 1). These two components describe the motion that broadly results from the LV translational movement (MV perpendicular radial motion fraction, pMr) and the LA related movements (MV centric radial motion fraction, cMr).

### Epicardial and intra-myocardial fat quantification

**Fig. 2:**
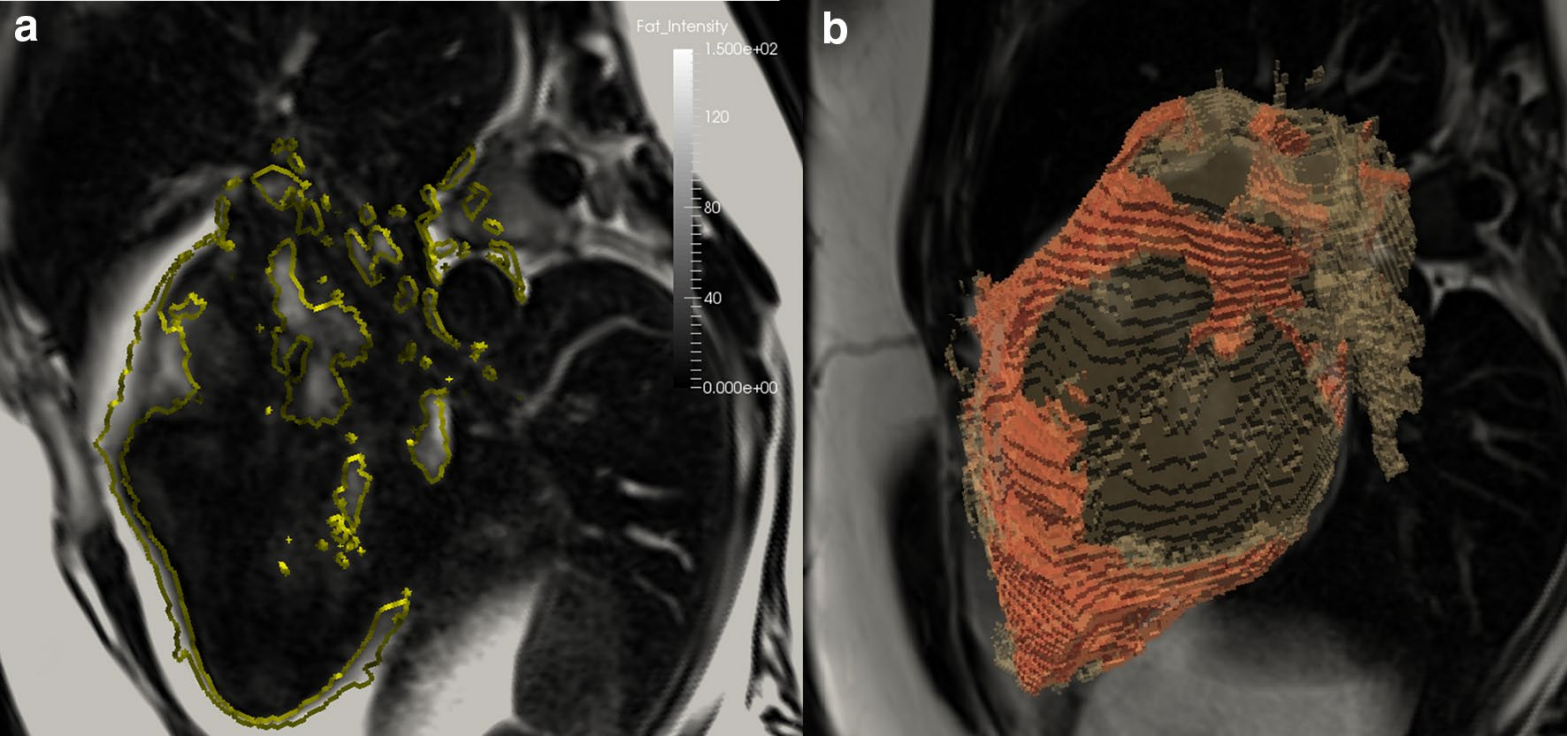
2D intra-myocardial fat fraction (**a**), and 3D Dixon adipose tissue quantification (**b**), fat (**a**) images from DIXON sequence. K-means segmentation of the heart and detection of the epicardial fat (**a**, **b**)

Fat measurement was performed following segmentation of the Dixon data into fat and water images (Fig. 2) [15]. Intra-myocardial fat was quantified using a hand-drawn ROI covering the myocardial septum in the 4CH view, with care taken to avoid partial volume effects (Fig. 2a). Epicardial fat was quantified blindly between patients and controls by segmenting cardiac volumes and Dixon fat images using Kmeans clustering by a semiautomatic program implemented in python and Paraview (Fig. 2). The volumes of the cluster corresponding to the adipose tissue were then calculated to define an absolute fat volume, which is then divided by the whole heart volume to obtain a 3D MRI Dixon adipose fraction. In order to normalize the volume of epicardial fat present by body size, absolute fat volume was indexed to BSA.

### Echocardiography

Diastolic function was assessed by echocardiography in patients using the American Society of Echocardiography guidelines [17]. The following parameters were collected—mitral inflow E and A waves, E′ lateral velocity of the MV annulus measured by tissue Doppler imaging, and aortic peak velocity and flow. One patient was excluded from the echocardiography analysis due to inadequate images secondary to the technical difficulty of the scan.

### Statistical analysis

Statistical analyses were carried out using SPSS Version 22 (IBM, Armonk, NY). All continuous variables are expressed as mean ± standard deviation (SD). Normality was checked using the Shapiro–Wilk test. Differences between groups were considered significant for p < 0.05. Intra-observer was assessed using intra class correlation coefficient (ICC). Groups were compared using the independent-samples *t* test for normally distributed continuous variables and the Mann–Whitney U test for non-normally distributed variables.

## Results

### Subject characteristics

Table 1-A summarises the demographics, global cardiac functional measures by CMR, and echocardiographic measurements in the ObT2D and control groups. There were no significant differences in age or gender between the ObT2D and control group. The average BMI in the ObT2D group classed them as morbidly obese (39 ± 3 kg/m^2^, range: 30–51 kg/m^2^); all individuals within the control group had a BMI < 30 kg/m^2^. At study entry, the ObT2D group had an average duration of T2Dof 11.4 ± 6.2 years and a mean HbA1c of 8.4 ± 1.1%.

**Table 1:** Demographic, clinical, echocardiographic and cardiovascular MRI measurements

	Healthy volunteers	ObT2D	HV/ObT2D
	(n = 19)	(n = 20)	p value
A. subject characteristics			
Age (years)	47.5 ± 12.5	54.6 ± 7.8	
Women (%)	45	40.0	
HbA1c (%)	–	8.5 ± 1.0	
Duration of diabetes (years)	–	11.3 ± 6.1	
Proportion of patients taking lipid-lowering therapy (%)	–	100.0	
Proportion of patients taking anti-hypertensives (%)	–	80.0	
History of CVD (%)	–	30.0	
Body surface area (m^2^)	1.9 ± 0.2	2.2 ± 0.2	**
Body mass index (kg/m^2^)	24.8 ± 2.6	39.7 ± 5.8	***
B. echocardiographic parameters in ObT2D patients			
Age (years)		54.6 ± 7.8	40–60
Mitral peak E velocity (m/s)		0.75 ± 0.24	0.75 ± 0.17
Mitral peak A velocity (m/s)		0.74 ± 0.18	0.62 ± 0.15
E/A ratio		0.99 ± 0.28	1.24 ± 0.39
E′ Lateral myocardium velocity (cm/s)		10.50 ± 2.4	12.5 ± 3.0
E/E′ ratio		7.6 ± 3.7	6.3 ± 2.2
Aortic valve peak velocity (m/s)		1.43 ± 0.57	
C. MRI LV function parameters			
Indexed LV mass (g/m^2^)	75.1 ± 14.9	80.8 ± 16.1	
LV EDV (mL)	122.8 ± 24.0	130.8 ± 34.0	
LV ESV (mL)	49.4 ± 13.4	44.8 ± 18.6	
Indexed LV EDV (mL/m^2^)	63.8 ± 11.4	58.9 ± 13.4	
Indexed LV ESV (mL/m^2^)	25.7 ± 7.1	20.1 ± 7.3	*
LV EF (%)	60.9 ± 7.4	66.1 ± 8.8	
Mean Mid. LV thickness (mm)	11.1 ± 2.0	12.9 ± 1.6	**

A. subject characteristics

B. echocardiographic LV function assessment in the ObT2D group

C. MRI LV function assessment in the control and ObT2D groups

Results are expressed as mean ± SD

* p < 0.05

** p < 0.01

*** p < 0.001

### LV functional assessment

Echocardiographic diastolic data for the ObT2D group are presented in Table 1-B. No echocardiographic data were obtained for the control group. Echocardiographic measures of diastolic function were within the normal standardised range in patients except E/E′ ratio, which was elevated at >8 in four subjects within the ObT2D group, and two greater than 15 (E/E′ range 8.3–17.1). Small significant differences in indexed LV ESV (p = 0.02) and mean mid myocardial LV thickness (p = 0.003) were found in the ObT2D group compared to control (Table 1-C) as compared to healthy volunteers.

### Left atrial size and strain measurements

Table 2 summarizes the MRI-derived LA volumetric and functional strain measurements. All MRI atrial volumetric measures were within the normal range for both the ObT2D and control groups. There were no significant differences in LA volumes or LVEF, even after indexing to BSA between the ObT2D and control groups.

**Table 2:** Left atrium volumes and functional parameters derived from MRI data

	Healthy volunteers	ObT2D	HV/ObT2D
	(n = 19)	(n = 20)	p value
LA EDV (mL)	87.1 ± 24.7	95.8 ± 22.3	
LA ESV (mL)	37.4 ± 14.7	40.5 ± 12.8	
Indexed LA EDV (mL/m^2^)	44.7 ± 9.8	43.3 ± 9.3	
Indexed LA ESV (mL/m^2^)	19.1 ± 6.1	18.2 ± 5.2	
LA EF (%)	57.7 ± 6.1	58.0 ± 7.9	
Longitudinal strain (%)			
Sl_R_	33.2 ± 6.8	29.4 ± 8.4	
Sl_C_	16.5 ± 4.8	13.1 ± 5.0	*
Sl_A_	16.7 ± 3.9	16.8 ± 4.8	
Sl_A_/SL_R_	0.5 ± 0.1	0.6 ± 0.1	
Longitudinal strain rate (%/s)			
SRl_S′_	1.4 ± 0.3	1.4 ± 0.4	
SRl_E′_	−1.5 ± 0.4	−1.2 ± 0.6	
SRl_A′_	−1.4 ± 0.4	−1.5 ± 0.5	
SRl_E′_/SRl_A′_	1.2 ± 0.5	0.9 ± 0.3	*
Radial motion fraction (%)			
Mr_R_	35.1 ± 6.7	31.5 ± 9.1	
Mr_C_	17.2 ± 4.7	13.6 ± 5.9	*
Mr_A_	17.9 ± 4.3	18.2 ± 4.3	
Mr_A_/Mr_R_	0.5 ± 0.1	0.6 ± 0.1	*
Radial relative velocity (%/s)			
Vr_S′_	1.4 ± 0.3	1.5 ± 0.6	
Vr_E′_	−1.6 ± 0.5	−1.3 ± 0.6	
Vr_A′_	−1.5 ± 0.5	−1.8 ± 0.6	
Vr_E′_/Vr_A′_	1.2 ± 0.5	0.7 ± 0.2	***
Decomposition of the radial motion fraction (%)			
MV centric cMr_R_	34.3 ± 9.6	37.2 ± 10.6	
MV centric cMr_C_	14.0 ± 5.7	11.3 ± 5.7	
MV centric cMr_A_	20.3 ± 5.6	26.0 ± 6.3	**
MV corrected perpendicular pMr_R_	53.3 ± 17.8	42.5 ± 12.6	*
MV corrected perpendicular pMr_C_	21.0 ± 15.2	14.0 ± 9.5	
MV corrected perpendicular pMr_A_	32.3 ± 10.7	28.6 ± 10.7	

Results are expressed as mean ± SD

* p < 0.05

*** p < 0.001

LA longitudinal strain (Sl_R_), longitudinal strain rate (SRl_S′_), and radial motion fraction (Mr_R_), were slightly *reduced* during reservoir phase in the ObT2D group compared to control and slightly *increased* for the atrial contraction phase (Sl_A_, SRl_A′_,Mr_A_). Both longitudinal strain and radial motion fraction were significantly reduced in the conduit phase (ObT2D vs. control, p = 0.04). Expressed as an E′/A′ ratio, there was a 28.5% reduction in the longitudinal strain rate ratio (SRl_E′_/SRl_A′_, ObT2D vs. control, p = 0.01). When radial motion was expressed as a relative velocity, the group difference was accentuated, with a 38.2% reduction in the Vr_E′_/Vr_A′_ ratio (ObT2D vs. control, p < 0.001). The radial motion ratio was also increased by 12.7% (Mr_A_/Mr_R_, ObT2D vs. control, p = 0.020).

In the ObT2D group, the MV corrected perpendicular component of the radial motion was decreased by 20% during the reservoir phase and by 33.6% in the conduit phase (ObT2D vs. control, p = 0.04 and p = 0.09 respectively). During the atrial relaxation (reservoir) and contraction phases, the MV centric radial components were higher in the ObT2D group compared to control (MV centric radial motion fraction in reservoir cMr_R_, p = ns; in LA contraction cMr_A_: p = 0.005). The increased cMr_A_ in the ObT2D group corresponded to a magnitude change of +28% relative to controls (20 vs. 26% in the MV centric radial plane defined in Fig. 1).

### Intra-myocardial and epicardial fat measurements

Intra-observer assessment resulted in an intra-class correlation coefficient of 0.95. Slight differences in intra-myocardial fat fraction were present between groups (ObT2D vs. control, 7.9 ± 3.7% vs. 6.0 ± 1.5%, p = 0.051; Table 3). In the ObT2D group, the epicardial fat measurement was increased by 69% for absolute volume and by 50% for indexed volume (ObT2D vs. control, 176.9 ± 76.6 vs. 298.9 ± 83.4 mL, p < 0.001 for absolute volume and 89.7 ± 29.6 vs. 134.6 ± 30.8 mL/m^2^, p < 0.001 for indexed volume). Following correction for BMI, these differences were attenuated but remained significant (p = 0.001).

**Table 3:** Fat measurements by MRI and echocardiography in healthy volunteers and obese patients

	Healthy volunteers	ObT2D	HV/ObT2D
	(n = 19)	(n = 20)	p value
Intra-myocardium dixon fat fraction (%)	7.9 ± 3.7	6.0 ± 1.5	
Dixon 3D epicardial fat volume (mL)	176.4 ± 68.6	273.8 ± 64.0	***
Indexed dixon 3D epicardial fat volume (mL/m^2^)	89.7 ± 25.2	123.6 ± 23.4	***
Dixon 3D epicardial fat fraction (%)	21.0 ± 4.0	28.4 ± 5.5	***

Results are expressed as mean ± SD

*** p < 0.001

### Correlations between left atrial function and measures of ObT2D

**Fig. 3:**
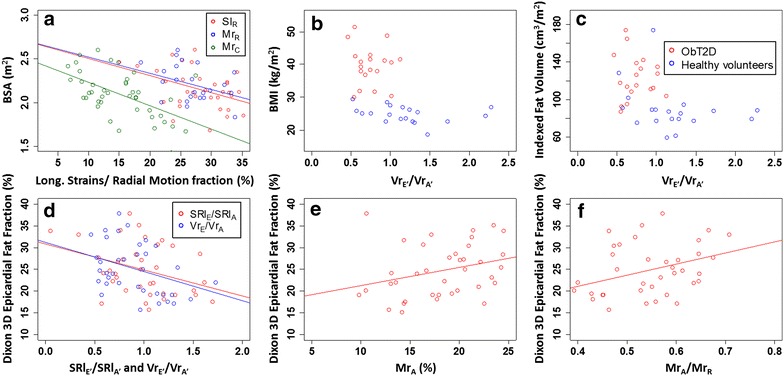
Correlations between BSA and LA functional indices in the control and ObT2D groups (**a**). Vr_E′_/Vr_A′_ related to BMI (**b**) and indexed adipose tissue volume (**c**). Correlations between LA functional indices and 3D Dixon cardiac fat fraction: longitudinal strain rates and radial relative velocities ratios (**d**), and radial motion fraction (**e**) and with radial motion fraction ratio (**f**)

Figure 3a–c illustrates the relationship between BSA and longitudinal strains and motion fractions (Sl_R_, Mr_R_ and Mr_C_, Panel a), Vr_E′_/Vr_A′_ and BMI (panel b), and Vr_E′_/Vr_A′_ and epicardial fat volume indexed to BSA (panel c). These demonstrated tight clustering of the Vr_E′_/Vr_A′_ values in the ObT2D group, with all of the subjects falling into the lowest two quartiles of Vr_E′_/Vr_A′_ (as defined using the normal cohort), and 70% within the lowest quartile of the normal range. There was no overlap in BMI and almost no overlap in epicardial fat. Significantly, all subjects with a BMI over 30 kg/m^2^ had low values of Vr_E′_/Vr_A′_– below or near 1 standard deviation from the control group average value. Other significant associations between Dixon 3D epicardial fat fraction and functional LA indices are presented in Fig. 3d–f (SRl_E_/SRl_A_, Vr_E_/Vr_A_, Mr_A_, Mr_A_/Mr_R_). Correlation coefficients between functional indexes and BSA or Dixon 3D epicardial fat fraction are reported Table 4.

**Table 4:** Correlations between functional indexes and body surface area and Dixon 3D epicardial fat fraction

	Body surface area (m^2^)		Dixon 3D epicardial fat fraction (%)	
	r	p	r	p
Longitudinal strain (%)				
Sl_R_	−0.561***	<0.001	−0.079	0.636
Sl_E_	−0.456**	0.004	−0.141	0.399
Sl_A_	−0.390*	0.014	0.090	0.591
Sl_A_/Sl_R_	−0.373*	0.019	−0.335*	0.040
Longitudinal strain rate (%/s)				
SRl_S′_	0.067	0.687	0.128	0.443
SRl_E′_	−0.302	0.062	0.043	0.798
SRl_A′_	0.394*	0.013	0.096	0.566
SRl_E′_/SRl_A′_	−0.006	0.971	−0.269	0.103
Radial motion fraction (%)				
Mr_R_	−0.570***	<0.001	−0.041	0.805
Mr_E_	−0.576***	<0.001	−0.143	0.390
Mr_A_	−0.284	0.080	0.153	0.359
Mr_A_/Mr_R_	0.278	0.087	0.244	0.140
Radial relative velocity (%/s)				
Vr_S′_	−0.064	0.698	0.252	0.127
Vr_E′_	0.437**	0.005	0.071	0.674
Vr_A′_	−0.024	0.884	−0.355*	0.029
Vr_E′_/Vr_A′_	−0.413**	0.009	−0.435**	0.006

* p < 0.05

** p < 0.01

*** p < 0.001

By univariate analysis, intra-myocardial fat was correlated to Vr_E′_/Vr_A′_ in the control group (r = −0.602; p = 0.006). No other significant correlations were seen in either controls or in the ObT2D group (correlation Vr_E′_/Vr_A′_ with intra-myocardial fat in patients r = 0.18; p = ns). Figure 3 highlights that there is a lack of dynamic range for Vr_E′_/Vr_A′_ in the ObT2D group, with values tightly clustered together. Therefore, linear correlation between BMI or epicardial fat was not performed to further analyse for any linear relationship. The ObT2D and control groups clearly did not form a normal distribution hence a pooled analysis was not performed.

The correlations between MV centric radial motion fraction and adipose tissue quantifications and patient biology were highly significant between cMr_R_ and BSA (r = −0.49, p = 0.002) and between cMr_R_ and BMI (r = −0.44, p = 0.005). Significant correlations were also found for indexed and non-indexed Dixon 3D epicardial fat volumes with cMr_R_ (r = −0.33, p = 0.04 and r = −0.41, p = 0.01, respectively) and pMr_A_ (r = 0.46, p = 0.004 and r = 0.37, p = 0.02). No significant correlations were present between atrial parameters and duration of diabetes or HbA1c.

## Discussion

Our data demonstrates that MRI-derived LA strain may be sensitive to early diastolic dysfunction. Our cohort of patients with obesity and T2D had an almost normal echocardiogram and non-enlarged LA volumes by standard imaging techniques [17]. Furthermore, our results show that LA strain is strongly correlated with BMI and epicardial fat supporting an association between adiposity and LA strain. If LA strain measures are validated, the ability to reliably detect early changes likely to be a precursor to clinically apparent disease may have important applications as both a clinical and research tool. Moreover, identification of individuals at risk for progression of the early diastolic dysfunction in ObT2Dmay enable (i) a more comprehensive characterisation of the patho physiology of early LA functional alteration in diastolic function, and (ii) testing of novel disease modifying strategies.

Alterations of LA function in the ObT2D population are considered to be secondary to pressure overload and are associated with poorer LV systolic function and higher LV mass [8]. Consistent with this, our results demonstrated an alteration of the LA function in individuals with obesity and T2D, as expressed by the ratio between conduit to LA contraction phase (E′/A′) and the LA contraction to reservoir phases. Both longitudinal strain rates SRl_E′_/SRl_A′_ and relative velocity Vr_E′_/Vr_A′_ ratios were significantly altered in the obese and diabetic group compared to matched controls, consistent with early stage diastolic dysfunction. Our data demonstrated a diminution of the conduit phase of LA function. A possible explanation for this finding could be elevated LV pressure, which is well described to be an early change in diastolic dysfunction. Coronary flow reserve has also been associated with LV filling pressure [18] in ObT2D which suggest a more sophisticated pathway of diastolic dysfunction. Additionally, the longitudinal (SRl_E′_/SRl_A′_) and radial (Vr_E′_/Vr_A′_) ratios depicted the change in the LA wall deformation, the passive conduit phase relative to the active phase of the LA. When radial motion was decomposed into cMr and pMr parts, the amplification of MV centric motion fraction during the LA contraction phase in the ObT2D group was consistent with the anticipated response of an early decrease in ventricular compliance impairing LV early filling. The observed reduction of reservoir MV corrected perpendicular motion fraction may related to LV contraction alterations as reported by Mochizuki et al. [19]. An increase in the functional parameters of LA contraction has been previously described in association with mild diastolic dysfunction, and has been proposed as a method of differentiating changes resulting from LV compliance as opposed to LV hypertrophy [8, 20].

An association between BMI and LA function has been previously described [21–23]. In our study, the use of MRI enabled the quantification of LA function, LV intra-myocardial and epicardial fat during the same examination. We demonstrated a clear relationship between LA relative velocity ratios and both BMI and epicardial fat volume. When the ObT2D and control groups were considered separately, there was no significant linear relationship between BMI or epicardial fat and LA function, perhaps due to our small sample size. This may suggest that LA functional alterations seen in the ObT2D group may occur over a certain threshold of epicardial adipose tissue volume or BMI. However, further data are required to test this hypothesis.

There are several proposed mechanisms by which the tissue alterations in obesity may influence the properties or mechanical load experienced by the LA. These include increased ventricular stiffness, inflammation mediated by adipokines, direct lipotoxicity or a metabolic effect mediated by insulin resistance [9]. Diastolic dysfunction has been previously shown to correlate with duration of diabetes, HbA1c and obesity [24]. Previous reports of correlations between strains and BSA or BMI were limited to ventricle [25] and detailed LV strain contraction analysis highlight the rule of glycemic control [26]. In our study, we did not find an association between LA strain and duration of diabetes or HbA1c, possibly due to the early stage of diastolic dysfunction detected. Prior echocardiographic data support a primary atrial myopathy in diabetes [27, 28]. Our study demonstrated alterations of conduit longitudinal strain and motion fraction, changes which are supportive of primary alterations of LV relaxation associated with mild diastolic dysfunction [29]. The strong correlation between MV centric motion fraction and BMI/epicardial fat might suggest MV centric motion fraction during LA contraction phase as a useful earlier predictor of diastolic dysfunction in the setting of obesity and T2D.

Our data demonstrated no significant differences in intra-myocardial Dixon fat between the ObT2D and control groups. A previous study reported a correlation between altered LV intra-myocardial fat fraction and LA function [10]. Our MRI data from subjects with early functional LA changes is a novel finding suggesting that intra-myocardial LV changes may not be a primary event driving very early dysfunction. The Dixon technique is only able to measure intra-myocardial fat *quantity*. It is insensitive to other myocardial tissue organisational alterations occurring in obesity and T2D. Further data regarding other ultra-structural analysis (e.g. from diffusion tensor imaging [30], T1 mapping [31], biochemical changes [32], or even electrophysiology [33]) may be helpful in extending the characterisation of LV structural changes in early diastolic dysfunction.

MRI is an attractive alternative to the routine use of echocardiography as it overcomes the practical problems of image attainment in obese patients and simultaneously characterises LA and LV functions as well as cardiac structure. The determination of cardiac fat volume by Dixon images has been previously reported [34]. However, our study is the first to use MRI to measure cardiac size, volume atrial strain and fat in one examination. This approach is likely to help to understand the pathogenesis of diastolic dysfunction in obesity and diabetes, and would enable testing of novel early therapeutic options in these patients. Further studies are needed to understand the different components of cardiac functional and structural modification in the setting of obesity and diabetes in order to explore possible therapeutic routes for reversal.

This study has several limitations, including the relatively small sample size, slightly age range difference between patients and healthy volunteers, and uniform diagnosis of obesity and diabetes, which did not permit deconvolution of the contributions of these conditions. Additional studies are required on the decomposition of radial motion into centric and perpendicular motions. Our clinical evaluation of the control subjects was also limited and measured blood pressures were not available, a clinical diagnosis of any cardiovascular disease including hypertension was excluded for all controls. The quality of the myocardial characterisation could be improved with the addition of T1 mapping or magnetic resonance spectroscopy data. In order to explore the nature of the early changes occurring in obesity and T2D, prospective studies are needed that include longitudinal imaging time points combined with objective clinical and metabolic measures (e.g. glucose tolerance test) of the progressive metabolic disorders over time. Although the control echocardiographic data was not available in this study, from a methodological perspective, it would be useful to directly compare diastolic assessment by echocardiography between the controls and ObT2D groups.

## Conclusion

MRI-derived strain measurements may be a useful tool to detect early abnormal LA function. Our cohort of patients with obesity and T2D had near normal echocardiograms and MRI-derived LV systolic function and volumetric measures of the LA and LV, and we demonstrated clear reductions in the conduit to LA contraction ratios (E/A) for longitudinal strain rate and radial motion fraction. Importantly these atrial measures correlated to both epicardial fat and to BMI. Further work in larger cohorts with a greater dynamic range of these parameters is required to understand the full implications of these findings.

## Abbreviations

CMR: cardiovascular magnetic resonance
cMr: MV centric radial motion fraction
EDV: LV end-diastolic
EFV: epicardial fat volume
ESV: end-systolic volumes
HFpEF: heart failure with preserved ejection fraction
ICC: intra-class correlation coefficient
LA: left atrial
LV: left ventricle
LVEF: LV ejection fraction
Mr_A_: LA contraction radial motion fraction
Mr_C_: conduit radial motion fraction
MRI: magnetic resonance imaging
Mr_R_: reservoir radial motion fraction
MV: mitral valve
ObT2D: obesity and type 2 diabetes
pMr: MV perpendicular radial motion fraction
Sl_R_: reservoir longitudinal strain
Sl_C_: conduit longitudinal strain
Sl_A_: LA contraction longitudinal strain
SRl_A′_: A′ longitudinal strain rates
SRl_E′_: E′ longitudinal strain rates
SRl_S′_: S′ longitudinal strain rates
SV: stroke volume
Vr_A′_: A′ radial relative velocities
Vr_E′_: E′ radial relative velocities
Vr_S′_: S′ radial relative velocities

## References

[1] Hogg K, Swedberg K, McMurray J (2004). Heart failure with preserved left ventricular systolic function: epidemiology, clinical characteristics, and prognosis. J Am Coll Cardiol.

[2] Gillebert TC, De Buyzere ML (2012). HFpEF, diastolic suction, and exercise. JACC Cardiovasc Imaging.

[3] Pirozzi F, Paglia A, Sasso L, Abete P, Carlomagno A, Tocchetti CG, Bonaduce D, Petretta M (2015). Mitral peak early diastolic filling velocity to deceleration time ratio as a predictor of prognosis in patients with chronic heart failure and preserved or reduced ejection fraction. J Geriatr Cardiol: JGC.

[4] Buffle E, Kramarz J, Elazar E, Aviram G, Ingbir M, Nesher N, Biner S, Keren G, Topilsky Y (2015). Added value of pulmonary venous flow Doppler assessment in patients with preserved ejection fraction and its contribution to the diastolic grading paradigm. Eur Heart J Cardiovasc Imaging.

[5] Borlaug BA, Paulus WJ (2011). Heart failure with preserved ejection fraction: pathophysiology, diagnosis, and treatment. Eur Heart J.

[6] Nanayakkara S, Kaye DM (2015). Management of heart failure with preserved ejection fraction: a review. Clin Ther.

[7] Kurt M, Wang J, Torre-Amione G, Nagueh SF (2009). Left atrial function in diastolic heart failure. Circ Cardiovasc Imaging.

[8] Santos ABS, Kraigher-Krainer E, Gupta DK, Claggett B, Zile MR, Pieske B, Voors AA, Lefkowitz M, Bransford T, Shi V (2014). Impaired left atrial function in heart failure with preserved ejection fraction. Eur J Heart Fail.

[9] Mookadam F, Goel R, Alharthi MS, Jiamsripong P, Cha S (2010). Epicardial fat and its association with cardiovascular risk: a cross-sectional observational study. Heart Views.

[10] Kilicaslan B, Ozdogan O, Aydin M, Dursun H, Susam I, Ertas F (2012). Increased epicardial fat thickness is associated with cardiac functional changes in healthy women. The Tohoku J Exp Med.

[11] Fox CS, Gona P, Hoffmann U, Porter SA, Salton CJ, Massaro JM, Levy D, Larson MG, D’Agostino RB, O’Donnell CJ (2009). Pericardial fat, intrathoracic fat, and measures of left ventricular structure and function: the Framingham Heart Study. Circulation.

[12] Evin M, Redheuil A, Soulat G, Perdrix L, Ashrafpoor G, Giron A, Lamy J, Defrance C, Roux C, Hatem SN (2016). Left atrial aging: a cardiac magnetic resonance feature tracking study. Am J Phys Heart Circ Physiol.

[13] Inoue YY, Alissa A, Khurram IM, Fukumoto K, Habibi M, Venkatesh BA, Zimmerman SL, Nazarian S, Berger RD, Calkins H (2015). Quantitative tissue-tracking cardiac magnetic resonance (CMR) of left atrial deformation and the risk of stroke in patients with atrial fibrillation. Am Heart J.

[14] Evin M, Cluzel P, Lamy J, Rosenbaum D, Kusmia S, Defrance C, Soulat G, Mousseaux E, Roux C, Clement K (2015). Assessment of left atrial function by MRI myocardial feature tracking. J Magn Res Imaging.

[15] Liu C-Y, Redheuil A, Ouwerkerk R, Lima JAC, Bluemke DA (2010). Myocardial fat quantification in humans: evaluation by two-point water-fat imaging and localized proton spectroscopy. Magn Res Med.

[16] Heiberg E, Sjögren J, Ugander M, Carlsson M, Engblom H, Arheden H (2010). Design and validation of segment—freely available software for cardiovascular image analysis. BMC Med Imaging.

[17] Nagueh SF, Bierig SM, Budoff MJ, Desai M, Dilsizian V, Eidem B, Goldstein SA, Hung J, Maron MS, Ommen SR (2011). American Society of Echocardiography clinical recommendations for multimodality cardiovascular imaging of patients with hypertrophic cardiomyopathy: endorsed by the American society of nuclear cardiology, society for cardiovascular magnetic resonance, and society of cardiovascular computed tomography. J Am Soc Echocardiogr.

[18] Kawata T, Daimon M, Miyazaki S, Ichikawa R, Maruyama M, Chiang S-J, Ito C, Sato F, Watada H, Daida H (2015). Coronary microvascular function is independently associated with left ventricular filling pressure in patients with type 2 diabetes mellitus. Cardiovasc Diabetol.

[19] Mochizuki Y, Tanaka H, Matsumoto K, Sano H, Toki H, Shimoura H, Ooka J, Sawa T, Motoji Y, Ryo K (2015). Clinical features of subclinical left ventricular systolic dysfunction in patients with diabetes mellitus. Cardiovasc Diabetol.

[20] Gabrielli L, Enríquez A, Córdova S, Yáñez F, Godoy I, Corbalán R (2012). Assessment of left atrial function in hypertrophic cardiomyopathy and athlete’s heart: a left atrial myocardial deformation study. Echocardiography.

[21] Lavie CJ, Amodeo C, Ventura HO, Messerli FH (1987). LEft atrial abnormalities indicating diastolic ventricular dysfunction in cardiopathy of obesity. Chest.

[22] Tsang TSM, Barnes ME, Miyasaka Y, Cha SS, Bailey KR, Verzosa GC, Seward JB, Gersh BJ (2008). Obesity as a risk factor for the progression of paroxysmal to permanent atrial fibrillation: a longitudinal cohort study of 21 years. Eur Heart J.

[23] Wierzbowska-Drabik K, Chrzanowski L, Kapusta A, Uznańska-Loch B, Płońska E, Krzemińska-Pakuła M, Kurpesa M, Rechciński T, Trzos E, Kasprzak JD (2013). Severe obesity impairs systolic and diastolic heart function—the significance of pulsed tissue Doppler, strain, and strain rate parameters. Echocardiography.

[24] Patil VC, Patil HV, Shah KB, Vasani JD, Shetty P (2011). Diastolic dysfunction in asymptomatic type 2 diabetes mellitus with normal systolic function. J Cardiovasc Dis Res.

[25] Enomoto M, Ishizu T, Seo Y, Yamamoto M, Suzuki H, Shimano H, Kawakami Y, Aonuma K (2015). Subendocardial systolic dysfunction in asymptomatic normotensive diabetic patients. Circ J.

[26] Cassidy S, Hallsworth K, Thoma C, MacGowan GA, Hollingsworth KG, Day CP, Taylor R, Jakovljevic DG, Trenell MI (2015). Cardiac structure and function are altered in type 2 diabetes and non-alcoholic fatty liver disease and associate with glycemic control. Cardiovasc Diabetol.

[27] Kadappu KK, Boyd A, Eshoo S, Haluska B, Yeo AET, Marwick TH, Thomas L (2012). Changes in left atrial volume in diabetes mellitus: more than diastolic dysfunction?. Eur Heart J Cardiovasc Imaging.

[28] Wang Y, Hou D, Ma R, Ding G, Yin L, Zhang M (2016). Early detection of left atrial energy loss and mechanics abnormalities in diabetic patients with normal left atrial size: a study combining vector flow mapping and tissue tracking echocardiography. Med Sci Monit.

[29] Guan Z, Zhang D, Huang R, Zhang F, Wang Q, Guo S (2010). Association of left atrial myocardial function with left ventricular diastolic dysfunction in subjects with preserved systolic function: a strain rate imaging study. Clin Cardiol.

[30] Sosnovik DE, Wang R, Dai G, Reese TG, Wedeen VJ (2009). Diffusion MR tractography of the heart. J Cardiovasc Magn Reson.

[31] Ling L-H, McLellan AJA, Taylor AJ, Iles LM, Ellims AH, Kumar S, Teh A, Lee G, Wong MCG, Azzopardi S (2014). Magnetic resonance post-contrast T1 mapping in the human atrium: validation and impact on clinical outcome after catheter ablation for atrial fibrillation. Heart Rhythm.

[32] Hudsmith LE, Neubauer S (2009). Magnetic resonance spectroscopy in myocardial disease. JACC Cardiovasc Imaging.

[33] Chao T-F, Lai Y-H, Yun C-H, Yen C-H, Wang K-L, Lin Y-J, Chang S-L, Lo L-W, Hu Y-F, Hung C-L (2014). The association between atrium electromechanical interval and pericardial fat. PLoS One.

[34] Homsi R, Meier-Schroers M, Gieseke J, Dabir D, Luetkens JA, Kuetting DL, Naehle CP, Marx C, Schild HH, Thomas DK (2016). 3D-Dixon MRI based volumetry of peri- and epicardial fat. Int J Cardiovasc Imaging.

